# From bench to bedside: current and future applications of molecular profiling in renal cell carcinoma

**DOI:** 10.1186/1476-4598-8-20

**Published:** 2009-03-17

**Authors:** Androu Arsanious, Georg A Bjarnason, George M Yousef

**Affiliations:** 1 Department of Laboratory Medicine, and the Keenan Research Centre in the Li Ka Shing Knowledge Institute. St. Michael's Hospital Toronto, Canada; 2 Department of Medical Oncology, Sunnybrook Odette Cancer Centre, University of Toronto, Canada; 3 Department of Laboratory Medicine and Pathobiology, University of Toronto, Toronto, Canada

## Abstract

Among the adult population, renal cell carcinoma (RCC) constitutes the most prevalent form of kidney neoplasm. Unfortunately, RCC is relatively asymptomatic and there are no tumor markers available for diagnostic, prognostic or predictive purposes. Molecular profiling, the global analysis of gene and protein expression profiles, is an emerging promising tool for new biomarker identification in RCC. In this review, we summarize the existing knowledge on RCC regarding clinical presentation, treatment options, and tumor marker status. We present a general overview of the more commonly used approaches for molecular profiling at the genomic, transcriptomic and proteomic levels. We also highlight the emerging role of molecular profiling as not only revolutionizing the process of new tumor marker discovery, but also for providing a better understanding of the pathogenesis of RCC that will pave the way towards new targeted therapy discovery. Furthermore, we discuss the spectrum of clinical applications of molecular profiling in RCC in the current literature. Finally, we highlight some of the potential challenging that faces the era of molecular profiling and its transition into clinical practice, and provide an insight about the future perspectives of molecular profiling in RCC.

## Renal cell carcinoma: A clinical overview

The American Cancer Society predicts that there will be about 54,000 new cases of kidney cancer in the United States in 2008, and about 13,010 people will die from this disease . Among the adult population, renal cell carcinoma (RCC) constitutes the most prevalent form of kidney neoplasia, and can be pathologically classified into subtypes: the clear cell type, which constitutes 80% of all cases, the papillary type, at around 15%, and the remaining 5% of other histological types. Certain subtypes, like chromophobe RCC, have a better prognosis compared to ccRCC. Other types, like collecting duct, medullary and sarcomatoid types have a more aggressive course. Early stage RCC is relatively asymptomatic, and the classical triad of flank pain, hematuria, and a renal mass only manifests very late in the course of the disease. The diagnoses of RCC is confirmed with imaging studies such as CT and ultrasound, and many cases of RCC are now accidentally discovered during routine imaging [1]. Kidney biopsy is an invasive technique that might result in complications and will not be able to provide accurate diagnosis in certain situations.

While surgery may be curative for localized disease, many patients eventually relapse. The 5-year survival rate for metastatic RCC is ≤ 10% [2,3]. The greatest risk of recurrence following resection of RCC is within the first 3–5 years [4]. Detecting recurrences early is important and can impact patient outcome since the likelihood of a favorable response to systemic treatment is greater when the metastatic burden is limited [5] and surgical resection of a single or limited number of metastases can result in long-term survival [6]. The anatomic extent of disease is the most consistent factor that determines prognosis in patients with resected RCC [7]. The UCLA Integrated Staging System (UISS) incorporates histologic grade and the ECOG performance status and has further improved on the prognostic information contained using the TNM system [8,9]. The most commonly used prognostic model for patients with metastatic disease is based on a multivariate analysis from the Memorial Sloan Kettering Cancer Centre [10].

While surgery is the treatment of choice for localized disease, treatment of advanced RCC is more challenging. Prior to the availability of targeted therapies, Interferon-α (INF) was the standard of care but was associated with a low response rate and significant toxicity [11,12]. High dose interleukin-2 (IL-2) has a similar response rate as IFN, but can cure approximately 3–5% of patients [13,14]. With targeted antiangiogenic drugs, we have entered a new era in the therapy of patients with advanced RCC [15,16]. In previously untreated patients Sutent improves overall survival when compared to INF [17] while Nexavar improves progression free survival (PFS) 2^nd ^line after immunotherapy when compared to best supportive care. Temsirolimus, an mTOR inhibitor, has been shown to improve overall survival vs. INF in previously untreated patients with high-risk RCC [18]. More recently the combination of Avastin and Interferon has been found to improve PFS when compared to Interferon alone in previously untreated patients [19,20] and Everolimus (RAD001), an orally administered inhibitor of mTOR, improves PFS in patients 2^nd ^line after progression on Sutent, Nexavar, or both compared to best supportive care [21]. Multiple other targeted drugs are in clinical trials.

## The current status of tumor markers in RCC

A tumor marker can be defined as a surrogate indicator that increases or decreases the clinician's suspicion to cancer susceptibility, onset, progression, or recurrence and whether a specific treatment will decrease the risk of such events [22]. There are currently no established tumor markers for RCC in clinical practice; tumor size and stage offer the only viable tools to predict prognosis [23]. More recently, a number of new molecular markers have been investigated, and although many show clinical potential, none has gained approved clinical application [24]. Lack of B7H1 and B7H4 expression is a strong predictor of overall survival in patients without metastases [25-28]. Another potentially important marker is IMP3[29,30].

While data from clinical trials provide general guidelines for the best 1^st ^and 2^nd ^line therapies for metastatic RCC, these are not always the best choices for each individual patient. There are very few biomarkers that can guide clinicians in the choice of therapy for each individual patient. In patients with clear cell RCC, responses to IL-2 were associated with the presence of alveolar features in more than 50 percent of the sample, and an absence of papillary features or granular features [31]. Carbonic anhydrase IX (CAIX) expression is HIF dependent and its expression is increased in VHL mutated RCC. High levels of CAIX expression are associated with a more favorable prognosis and a greater likelihood of a response to IL-2 [32,33]. There are no biomarkers available to predicting responsiveness to molecularly targeted agents. Measurements of VEGF and the soluble VEGF receptor do change in response to treatment but whether such alterations can be used, as a surrogate for tumor responsiveness remains unknown.

## Molecular profiling

Molecular Profiling (MP) can be defined as the classification of biological specimens, like tissues, blood or urine, based on multiple molecule (like gene, protein, miRNA) expression patterns or genomic changes for diagnostic, prognostic, and predictive purposes [34]. The 1990s ushered in an era of information churning out faster than its analysis. The completion of the Human Genome Project, rapid advances in bioinformatics, the application of new technologies like mass spectroscopy and array analysis – that allow simultaneous high throughput analysis of thousands of molecules – and emergence of new treatment options like targeted therapy, necessitated the birth of global analysis to allow for a more complete understanding of the malignant process. MP creates a paradigm shift from the traditional approach of looking at one molecule a time to the simultaneous high-throughput analysis of thousands of molecules. The focus starts to switch into a more "global" analysis of dysregulated genes and proteins, and other molecules, in order to obtain a better understanding of the potential "cross-talks" between them. This has substantial clinical impact in the field of clinical oncology, as described below in more details with specific reference to RCC.

## Molecular profiling approaches

Molecular profiling is a multifaceted process which can be explored on three different levels: genomic, transcriptomic and proteomic. Each takes a different angle on the global picture. Table 1 summarizes the different common tools for molecular profiling. Among the tools mentioned, the most widespread are microarray technology and mass spectrometry.

**Table 1:** Different tools for molecular profiling at the genomic, transcriptomic, and protein levels

Genomic
• Comparative Genomic Hybridization (CGH)
• Array-based CGH
• Single Nucleotide Pleomorphism (SNP)
• Multi-colour FISH
• high-throughput sequencing techniques (hybridization-based, cycle-based, and single molecule based)
• High-throughput analysis of methylation.
• Spectral karyotyping (SKY)

Transcriptomic

• Microarray
• mRNA
• microRNA
• Serial Analysis of Gene Expression (SAGE)
• Expressed Sequence Tags (EST)
• Digital Differential Display (DDD)
• Single Nucleotide Pleomorphism (SNP)
• Quantitative RT-PCR
• High throughout sequencing
• In-situ hybridization

Proteomic

• Mass spectrometry (different versions)
• Protein microarray
• Tissue microarray
• Chromatography

## Microarray analysis

Since its introduction in the mid-1990s [35], microarray analysis has become an established technique to simultaneously compare gene expression patterns between different conditions. Generally speaking, a microarray is a compact chip containing a large number of well-defined immobilized capture molecules (synthetic oligos, mRNA transcripts, proteins, antibodies etc), that are capable of assaying molecules through hybridization with a labelled probe [36]. It has been used to analyze gene expression profiles in many malignancies through exploration of the alterations that account for the transition from a benign, to a dysplastic, to an invasive cancer, and of alterations leading to the development of metastasis. Microarrays have been of great value in the discovery of biomarkers for the field of diagnostic pathology, which have been discussed elsewhere [37]. As shown in table 1, a microarray can compare different entities among themselves, whether genomic, transcriptomic, or proteomic. mRNA microarray is the most popular approach. Databases of gene expression profiles in various malignancies are now publicly available [38]. More recently, the technology extended to include microRNA microarrays, DNA arrays (comparative genomic hybridization; CGH), protein arrays, and tissue microarrays. There are several platforms for microarray analysis, including planar, immobilized bead, liquid bead, and barcode nanoparticles or quantum dots.

Advantages of microarray technology include the minimal amount of tissue and reactants needed to generate feasible results and the high degree of sensitivity. It can be also automated and can produce quantitative data. Although microarray is continually being improved, many drawbacks need to be addressed before bringing the technology to the bedside, including the lack of standardization, reproducibility, variability of the results due to specimen type and preparation, and the need to develop a quality control for the procedure. Added to this is the microarray's inability to reveal post-transcriptional gene control [39]. More details about microarray are reviewed elsewhere [40].

## Mass spectrometry

Further insight into the molecular mechanisms of cancer can also be gleaned through proteome analysis. Proteomics offer considerable advantages over its genomic counterpart since protein is the ultimate agent responsible for the malignant phenotype. Proteomics can also identify alterations in post-translational modifications and cellular trafficking that may not be detected by RNA-based expression studies. Mass spectrometry (MS) has proven to be an invaluable tool for the characterization of protein structure and their amino-acid sequences. MS promises to unveil the complex molecular events characterizing tumorigenesis and help in the study dynamic protein expression, post-translational modifications, cellular and sub-cellular protein distribution, and protein-protein interactions, which has already culminated in the identification of many cancer-related biomarkers and potential new drug targets [41].

Several techniques have been used for protein profiling. Two-dimensional (2-D) gel electrophoresis coupled with MS was the traditional strategy and, to date, has yielded several potentially relevant cancer biomarkers [42]. Other methods include solid-phase extraction followed by matrix-assisted laser desorption/ionization mass spectrometry (MALDI MS) as well as selective surface binding and surface-enhanced laser desorption/ionization (SELDI MS) [43]. More recently, another approach has been developed in which proteins from two samples to be compared are tagged with differing isotopic composition. The two samples are then combined and processed in a single batch thereby allowing relative quantification to be performed. An effective labeling strategy uses isotope-coded affinity tag (ICAT) [44], or, in the most recent variation, uses isobaric tagging reagent, iTRAQ [45], followed by multidimensional LC and MS/MS analysis which allows simultaneous quantification. Recent guidelines from the National Academy of Clinical Biochemistry highlighted the need for standardization and quality control before MS can be involved in clinical care [46]. Detailed discussion about MS is beyond the scope of this review.

Traditionally, the goal of most proteomic studies is to identify biomarkers that can be measured by enzyme-linked immunosorbent assay (ELISA). Improvements in proteomic technology may be changing this because there are now efforts to develop proteomic technologies directly into clinical diagnostic tests. An example of this technology is surface-enhanced laser desorption ionization time-of-flight (SELDI-TOF) MS. This technology, combined with pattern recognition based on bioinformatics tools, and discriminatory spectrum proteomic profiles can be generated to help discriminate individuals with cancer from those with benign disease.

## Other techniques

Comparative Genomic Hybridization (CGH) is a method that allows for the comparison of genomic alterations as DNA sequence copy variations, insertions, and deletions, between two types of tissues. Chromosomal changes in cancer can be scanned using CGH whereby the test (cancer) and control (normal), are labeled and hybridized with normal metaphase chromosomes. Competition for hybridization with the metaphase chromosomes arises between test and control DNA and fluorescent techniques are then used to assess DNA gain or loss in cancer [47]. A more recent advance in the technique utilizes a microarray format that allows much better resolution in detecting chromosomal aberrations.

Another technique assays genomic changes using Single Nucleotide Polymorphism (SNP), which are variations in a given DNA base between different members of the same species. Found at frequencies of one every 1000–2000 base pairs, much of human genetic diversity is attributed to SNP variation between individuals. This gives SNP analysis a potentially useful diagnostic application for haplotype-related disease, and recent work has begun taking advantage of this [48].

Recent evidence suggests that microRNAs, small non-coding oligonucleotides that regulate gene expression, are dysregulated in various malignancies, and have promising clinical roles as cancer biomarkers. miRNA microarrays have shown the ability to accurately classify cancers and to be potential prognostic and predictive markers for many tumors [49].

## Clinical utility of molecular profiling

The suite of clinical applications of molecular profiling in cancer is broad, encompassing a wide variety of fields. Constituting some of these clinical applications are diagnosis, prognosis, prediction of treatment efficiency, patient follow-up after surgery for early detection of recurrence, and the sub-grouping of patients into smaller categories, thus allowing for individualization of treatment options. One of the revolutionary aspects of MP is changing our traditional paradigm in classifying cancer. Pathological classification can shift from the histological scale – that often gives little information on prognosis, individualized treatment options, and chance of recurrence, overlooking the fact that many patients with similar histological types might experience markedly different disease courses – to the molecular scale, which offers a highly detailed, global perspective on the disease process, promising superior performance over traditional classifications.

Moreover, MP is a key for better understanding of cancer initiation and progression. MP, which can also lead to the development of new targeted therapy options – especially ones designed against the inherently intractable metastatic stages of cancer – that can complement existing treatment options. Simultaneous analysis of multiple markers identified by MP can lead to much improvement in sensitivity and specificity. Another interesting application is the ability of a MP signature to distinguish benign from malignant tumors, which is not always feasible via histological analysis alone. Tumors of unknown origin are another common challenge in histopathology practice, and the use of MP signatures to identify the tissue of origin in these poorly differentiated tumors, where morphology cannot help, will have a great impact on patient care.

## Molecular profiling in RCC

A more detailed look at how MP can affect our understanding and management of RCC will now be discussed. Molecular profiling of RCC has been performed at different levels, including RNA, protein, genomic and more recently miRNA. As is the case in other tumors, there are several potential clinical applications of MP in RCC.

The first application is investigating the presence of a "signature expression profile" in RCC that would allow a distinction to be made between it and normal tissue. A number of studies have analyzed differential gene expressions in RCC at the mRNA level [50-54]. Lenburg *et al. *[55] highlighted the poor overlap among many of these studies and underscored the need for accurate statistical methods to be applied to microarray analysis and also to filter out defective samples and genes that are not reliably detected. Liou et al [56] demonstrated a significant difference between data obtained from tissues vs. cell lines. When Laser microdisected tissues of RCC were used for microarray analysis, the top dysregulated genes identified were significantly different from bulk tissues [57], suggesting that a more "pure" malignant population can lead to more accurate results. Dalgin et al recently identified a number of hypermethylated genes in RCC using methylation assays coupled with computational screening [58].

There are a few reports on protein profiling of RCC [59-63] that have identified a number of potential biomarkers [64,65]. Urinary proteomics, and more recently metabolomics, are emerging new fields for biomarker discovery in urinary tract diseases [66]. In kidney cancer, a recent pilot study analyzed urine samples from RCC and controls [67], where metabolic profiling and pathway analyses were significantly different. Another study investigated the clinical utility of SELDI profiling of urine samples in conjunction with neural-network analysis to detect renal cancer and to identify proteins of potential use as prognostic markers, but the results were not reproducible [68]. Identifying proteomic markers directly from the serum of RCC patients is more challenging. Attempts of serum profiling of RCC patients by SELDI-TOF [69,70] were not reproducible when validated in an independent population [71].

Very recently, miRNA research has emerged with great clinical potential in RCC. Potential usefulness of miRNA profiling in RCC include its potential ability to determine the tissue of origin (through a kidney-specific signature) in tumors of unknown primary [72]. A recent study identified four miRNAs that were significantly up regulated in kidney cancer [73]. More recently, a total of 33 differentially expressed miRNAs were identified in clear cell RCC, including 21 up-regulated miRNAs (our data, submitted for publications). Bioinformatics and literature searches showed that many of these have been reported to be dysregulated in other malignancies and have a potential role in cancer pathogenesis. Interestingly, the differentially expressed miRNAs showed a significant correlation with reported regions of chromosomal aberration sites that included regions of amplification or loss. Preliminary analyses showed that some of these targets can be directly involved in RCC pathogenesis (our manuscript, submitted for publication).

A second interesting potential role for MP is distinguishing the different types of renal tumors. A good example of this is the differentiation between oncocytoma and chromophobe RCC – two different forms of kidney tumors notoriously confused for one another because of their microscopic similarity. Indeed, both cancers were found by microarray to constitute a high degree of similarity in mitochondrial gene expression. Further gene analyses, however, showed differences in gene expression profiles between the two conditions [74]. Another study used mRNA expression profiles to properly distinguish between clear cell carcinoma and chromophobe carcinomas [75]. A third report showed the reliability of MP in accurately classifying different subtypes of RCC [76].

Approximately 5% of clear cell renal cell carcinomas contain a sarcomatoid component. The nature of this component is not well understood. Studies, however, have begun shedding light on this topic through MP. Comparing allelic loss patterns in clear cell and sarcomatoid components of RCC, Jones et al [77] suggested that both components are derived from the same progenitor cell. Different patterns of allelic loss were observed in clear cell and sarcomatoid components from the same patient, indicating genetic divergence during the clonal evolution of RCC. Moreover, retrospective analysis has shown superior performance of MP in detecting mixed subtypes and cases with confusing histological patterns. Another report identified groups of genes that can distinguish the clear cell and chormophobe types of RCC [78]. Higgins et al. [79] used DNA microarrays to classify, on a molecular scale, papillary carcinomas from conventional RCC and cancers from different parts of the kidney.

Monzon et al [80] recently showed that SNP arrays can detect characteristic chromosomal aberrations in paraffin-embedded renal tumors, and thus offer a high-resolution, genome-wide method that can be used as an ancillary study for classification and potentially for prognostic stratification of these tumors. Using microarray analysis, gene signatures were identified that distinguish RCC from other cancers with 100% accuracy. Differentially expressed genes during early tumor formation and tumor progression to metastatic RCC were also found. Moreover, a previously described "global" metastatic signature was validated in RCC. [81]. Another study identified a set of 80 genes that was sufficient to classify tumors with a very low error rate. Distinct gene expression signatures were associated with chromosomal abnormalities of tumor cells, metastasis formation, and patient survival. [82]. Such studies underscore the practical usefulness of MP in determining the nature and subtype of the patient's illness.

Molecular profiling has important prognostic applications in RCC. The use of microarrays identified numerous prognostic biomarkers. Such markers can help stratify patients into prognostic risk groups and guide future therapy decisions. A recent microarray analysis identified two major subgroups within RCC, based on gene expression profiling, that differ in biological behaviour despite similarity in histology [83]. Another microarray-based analysis has shown that approximately 40 genes can accurately make the distinction between patients with a relatively non-aggressive form of the disease compared to patients with aggressive disease [84]. These molecular signatures were shown to supersede conventional staging in predicting outcome. Moch et al [85] identified 89 differentially expressed genes in RCC. One of these, vimentin, is a marker of poor prognosis.

In addition to expression profiling, cytogenetic changes might also have prognostic value in RCC. A recent report showed that loss of chromosome 9p was found to be an independent indicator of poor prognosis in RCC [86]. Boer et al found that gene expression signature can distinguish early from advanced metastatic stage (Stage IV) tumors [87].

Using tissue microarray analysis, Kim et al [88] constructed a combined molecular and clinical prognostic model for survival that was significantly more accurate than standard clinical parameters. The recent identification and potential incorporation of molecular markers into current staging systems of renal cell carcinoma is expected to revolutionize the staging of the tumor [89]. Two prognostic nomogram models to predict survival after nephrectomy were created. One was based exclusively on molecular markers and the other on a combination of clinical variables and molecular markers [90]. Findings suggest that the integration of molecular profiling with clinical parameters could enhance diagnosis and prognosis of the disease.

A forth important application of MP in RCC is identifying predictive markers. It can be used to predict response to immunotherapy and targeted therapy [91]. Profiling analysis can be very helpful in identifying targets for immunotherapy and targeted molecular therapy [92]. The fifth and critical objective of MP is elucidating the pathogenesis of RCC. Accumulation of hundreds of dysregulated genes identified by different studies elicited the next step of "understanding" the interaction between these molecules. An early report, using Gene Ontology analysis, identified a number of up and down regulated biological processes, some overlapping with other malignancies and others are unique for RCC [93]. Similar findings were observed by Gieseg et al [94], who identified enrichment of certain biological processes like cellular adhesion, matrix integrity, and signal transduction mechanisms. Liou et al found that genes involved in cell adhesion where dominantly upregulated while those involved in transport were down regulated [95].

More recently, pathway analyses have emerged. Extensive pathway analysis allowed the discovery of significant pathways in clear cell RCC, including glycolysis, propanoate metabolism, pyruvate metabolism, the urea cycle, and arginine/proline metabolism, as well as in the non-metabolic p53 and FAS pathways [96]. Knowledge of networks, processes, and pathways altered in kidney cancer may be used to influence the choice of therapy. More recently, we identified a number of pathways that are significantly enriched in RCC. While some of these are "commonly" dysregulated pathways in many cancers, like cell cycle, apoptosis, cell adhesion and MAP kinase pathways, other interesting pathways, not previously linked to RCC were also ientified, including insulin signaling, PPAR signaling, hemostasis and blood coagulation, pyruvate metabolism and TCA cycle (our manuscript, submitted for publication) are also involved. Interestingly, although there was only a minimal overlap between published protein lists, there was significant overlap between the identified pathways between groups. Preliminary analysis also shows the presence of interaction networks among dysregulated proteins [96].

More recent efforts are focused on integrated analysis of different levels of molecular changes to allow better understanding of the pathogenesis of RCC. A recent report performed an integrated analysis of DNA and RNA profiles of RCC samples. Combining genomic and transcriptomic data, they identified 71 differentially expressed genes in aberrant chromosomal regions and observed, in amplified regions, a predominance of up-regulated genes and a trend to clustering [97].

Cytogenetic analysis has also been an invaluable tool to insight into the pathogenesis of RCC. Earlier studies showed that chromosomal aberration are involved in the development of RCC, and that they can guide our understanding of the molecular events needed for development and progression of RCC [98,99]. A study showed that array-based CGH is capable of distinguishing the vast majority of renal cell carcinomas from normal and benign lesions based on their genetic profiles of DNA copy number variations [100]. Yoshimoto et al., also using array CGH, found that chromosomal alterations in clear cell RCC are significantly different from those of chromophobe RCC, and that up and down-regulated genes significantly localize within areas of chromosomal gain or loss, respectively [101].

## Challenges of molecular profiling

The transition of molecular profiling from the research bench into a clinical setting necessitates addressing several challenges. One of these is how to integrate several modalities to achieve a multi-dimensional molecular profile of the patient's specimen. This requires a collaborative effort between many elements of the health care team, particularly clinicians, research scientists, computer experts, and statisticians. A team approach is necessary for the transition of various parameters into a clinically meaningful format that will help in obtaining a comprehensive picture of each individual tumor and aid in diagnosis, assist in prognosis, and in individualizing the line of treatment. Accumulation of data from various research laboratories and meta analysis studies will definitely help to reach a more solid understanding of how to transfer MP into a clinical setting. Full transparency in reporting results (especially the negative ones) should be emphasized to avoid selection bias for positive results reporting.

Challenges facing the use of MP in clinical decision making have been addressed in details in a number of recent excellent reviews [102]. A major limitation of most published reports is the heterogeneity of the analyzed material, from tissues to cell lines to biological fluids, and combining different histological types, stages and grades. An important issue is the need for standardization of MP testing. Standardization encompasses several aspects including the specimen type (fresh frozen versus formalin fixed paraffin embedded tissues), the appropriate method of specimen storage, the platform to be used, the experimental conditions, and the clinical interpretation of the test results. Another important issue is the choice of the targets (which and how many genes or proteins to be included).

Tissue preservation and handling is a prime issue to be considered. Formalin-fixed tissue used for histopathologic diagnosis cannot be used for MP. Sacrificing a portion of the diagnostic tissue for molecular analysis might compromise the quality of the pathological diagnosis. Ongoing solutions for this problem include the use of non-formalin alcohol based fixatives which preserve RNA quality.

Heterogeneity of the tumor tissue is another important factor to be considered in this regard. Tumor tissue represents a mixture of tumor, adjacent normal, and stromal elements. There are different approaches that have been taken to deal with this problem, including a cultured cell line, global survey, and micro-dissection profiling [103].

A new molecular profiling analysis test must be able to provide additional information for diagnostic, prognostic, or predictive purposes that are beyond classical factors. Unfortunately, due to lack of prospective studies, the performance of many molecular profiling experiments are not additionally significant than classical markers. Moreover, at the discovery phase, many of the experiments lack statistical significance since they are not initially designed with enough power to address the hypothesis. Added to this is the lack of well-defined clinically annotated cases and specimens. Results must therefore be validated, preferably by an independent data set. Cross-validation within the same set is a weakness that hinders generalization of the results.

A recent report by the National Academy of Clinical Biochemistry Laboratory Medicine Practice Guidelines [104] expounded upon two main technologies commonly implemented in MP, microarray and mass spectrometry, and developed recommendations on what must be done before for their application in the clinical realm.

Cost is another challenge. This includes running costs and the need to buy new expensive equipments for molecular testing. However, the cost of the commonly used techniques, such as microarrays, continues to decrease as it becomes more widespread. In addition, focusing on fewer targets will be an important factor in reducing costs. Ethical and legal issues are expected to represent an additional challenge especially in cases of hereditary or familial tumors.

## Molecular profiling: A glimpse at the future

Before the era of molecular profiling, cancer diagnosis, prognosis, and subsequent treatment decisions were based on histopathologic parameters, usually the tissue of origin and the stage and grade of the tumor. Years of experience have shown morphological classification to be deficient in many aspects and that patients with the same histopathologic picture can have unexplained variable outcomes. Individual molecular markers have been slowly added to ameliorate the accuracy of predicting prognosis and prediction of treatment efficiency. Examples of this are the immunohistochemical assessment of the estrogen and progesterone receptors in breast cancer and pre-operative prostate specific antigen (PSA) measurement.

Entering into the era of molecular profiling, many scientists were excited and felt that MP would be able to revolutionize our clinical practice and replace most traditional tools [105]. After a period of initial enthusiasm, scientists and clinicians began to realize the major obstacles that face the clinical utilization of MP. Molecular profiling is not likely to replace anatomical pathology, and a more stance is that it will slowly be added in conjunction with the classical diagnostic and prognostic parameters.

Development of clinically meaningful application of molecular profiling can be roughly divided into three stages [106]. The first stage, nearly accomplished, is the identification of all the "players" that share in the pathogenesis of cancer. With completion of the human genome project and the major advances in gene prediction programs, many new genes, splice variants, and non-coding molecules, have been identified. This sets the stage for the next phase of comparing molecular profiling in normal vs. cancer and at different stages of cancer. Data is now piling about differential gene and protein expression in renal cell carcinoma. Cytogenetic and microRNA changes are also accumulating.

An emerging important, and more difficult, third stage is the incorporation of these multiple parameters into one picture. The ongoing efforts of protein-protein interactions analysis and pathway analysis represent two important steps on the right track towards an understanding of the meaning of these pathological changes, and consequently applying them for diagnostic and treatment efforts. At this phase, bioinformatics will play a chief role. The integral task of bioinformatics encompasses a wide variety of areas including the availability of cancer databases and providing sophisticated analytical tools that are capable of analyzing an enormous amount of data.

MP can also provide a "multi-parametric" approach in cancer biomarkers, where a combination of multiple markers will lead to enhanced sensitivity and specificity, as compared to individual markers. More recently, we have witnessed the emergence of commercial molecular profiling-based analytical tests that are used to answer specific questions related to certain subgroups of patients. One commercially available kit is used to assess the chance of recurrence in certain subgroups of breast cancer patients. Another kit is utilizes gene expression profiling to identify the tissue of origin in cancers of unknown primary. Finally, the molecular profiling approaches should be customized for different cancers. The questions to be addressed and the clinical utility depend on prevalence, natural history, and the available treatment tools for each individual cancer.

**Figure 1:**
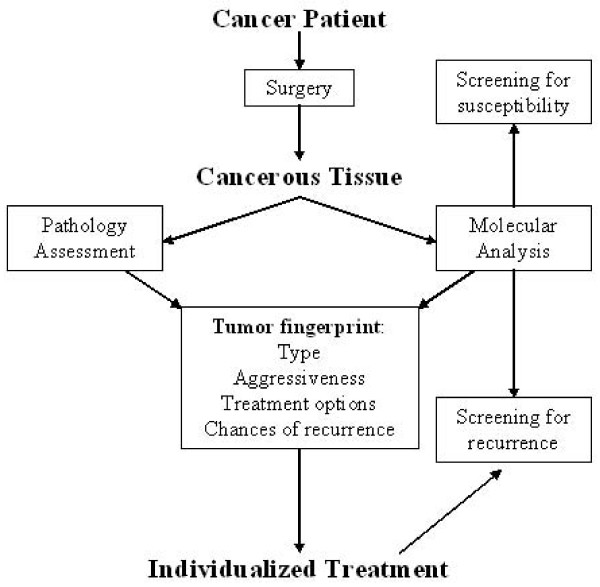
**A possible scenario of how molecular profiling can be integrated with clinical decision making for kidney cancer patients**.

Figure 1 shows one possible future scenario where molecular analysis is performed hand in hand with usual histopathologic evaluation, allowing for a more individualized picture that constitutes more details about every individual cancer, including the aggressiveness and treatment options. This tumor "fingerprint" will help avoiding extra costs and side effects associated with the unnecessary use of certain lines of treatment in patient who will not benefit from them.

## Non-standard abbreviations

RCC: Renal Cell Carcinoma; MP: Molecular Profiling; MS: Mass Spectrometry.

## Competing interests

The authors declare that they have no competing interests.

## Authors' contributions

AA drafted and wrote the paper, designed the tables and the figures. GB contributed to the clinical utility and molecular profiling in RCC section. GY corrected and finalized the manuscript. Both authors read and approved the final manuscript.
